# How does the correction in lumbar lordosis affect the spinopelvic realignments in degenerative lumbar scoliosis underwent scoliosis surgery?

**DOI:** 10.1186/s40001-023-01339-5

**Published:** 2023-10-05

**Authors:** Zifang Zhang, Jianing Song, Shu Jia, Zhikang Tian, Zhenyu Zhang, Guoquan Zheng, Chunyang Meng, Nianhu Li

**Affiliations:** 1 Affiliated Hospital of Jining Medical University, Jining Medical University, Guhuai Road 89, Jining, 272007 China; 2 Department of Orthopedics, Shandong University of Traditional Chinese Medicine, Jingshi Road 16369, Jinan, 250014 China; 3 Beijing Rehabilitation Hospital, Capital Medical University, Beijing, China; 4 The Spine Surgery, The first medical center of the Chinese PLA General Hospital, Beijing, China

**Keywords:** Degenerative lumbar scoliosis, Lumbar lordosis, Spinopelvic alignment, Quality of life

## Abstract

**Background:**

To evaluate the effects of correction in lumbar lordosis (LL) that have on full-body realignments in patients with degenerative lumbar scoliosis (DLS) who had undergone long sacroiliac fusion surgery.

**Methods:**

A multi-center retrospective study including 88 DLS patients underwent the surgical procedure of long sacroiliac fusion with instrumentations was performed. Comparisons of radiographic and quality-of-life (QoL) data among that at the pre-operation, the 3rd month and the final follow-up were performed. The correlations between the LL correction and the changes in other spinopelvic parameters were explored using Pearson-correlation linear analysis and linear regression analysis. The correlation coefficient (*r*) and the adjusted *r*^*2*^ were calculated subsequently.

**Results:**

All radiographic and QoL data improved significantly (*P* < 0.001) after the surgical treatments. The LL correction correlated (*P* < 0.001) with the changes in the sacral slope (SS, *r* = 0.698), pelvic tilt (PT, *r* = -0.635), sagittal vertical axis (SVA, *r* = −0.591), T1 pelvic angle (TPA, *r* = −0.782), and the mismatch of pelvic incidence minus lumbar lordosis (PI–LL, *r* = −0.936), respectively. Moreover, LL increased by 1° for each of the following spinopelvic parameter changes (*P* < 0.001): 2.62° for SS (*r*^*2*^ = 0.488), −4.01° for PT (*r*^*2*^ = 0.404), −4.86° for TPA (*r*^*2*^ = 0.612), −2.08° for the PI–LL (*r*^*2*^ = 0.876) and -15.74 mm for SVA (*r*^*2*^ = 0.349). Changes in the thoracic kyphosis (*r* = 0.259) and pelvic femur angle (*r* = 0.12) were independent of the LL correction, respectively.

**Conclusions:**

LL correction correlated significantly to the changes in spinopelvic parameters; however, those independent variables including the thoracic spine and hip variables probably be remodeled themselves to maintain the full-body balance in DLS patients underwent the correction surgery.

## Background

The prevalence of degenerative lumbar scoliosis (DLS) is very common, ranging from 32% to 68% [1–4]. Full-body imbalance often coexists with neurological dysfunction in those DLS patients [5]. It was reported that the loss of lumbar lordosis (LL) can be considered as the initiating event of sagittal imbalance, which would push the center of gravity forward in such patients [6]. The sagittal malalignment is compensated for by the parts of axial skeleton in which thoracic kyphosis, pelvic tilt, and knee flexion increase, according to the grade of malalignment required to maintain a standing posture with a horizontal gaze [7, 8]. As a result, an ideal correction in LL must be performed to restore full-body balance for those DLS patients [9]. Furthermore, previous studies have illustrated that the surgical procedure of thoracolumbar fusion with instrumentations can restore the spinopelvic alignments effectively in DLS [4, 10, 11]. However, the abnormal correction in LL may result in abnormal spinopelvic alignments, which would increase the incidence of mechanism complications, and deteriorate the QoL accordingly [12–17] because of the mismatch among the spine, pelvis and lower extremities [12, 18].

As a result, it is essential for spinal surgeons to recognize the associations of the LL correction with the changes in other spinopelvic parameters in evaluation and management of DLS patients, which have been seldomly reported in previous studies although. Therefore, we performed this current study to investigate the effects of LL correction that have on spinopelvic realignments in DLS patients who had undergone the surgical procedure of long sacroiliac fusion with instrumentations.

## Methods

### Patients data

This is a multi-center observational study. The Ethics committee of the Shandong University of Traditional Chinese Medicine, the affiliated hospital of Jining Medical University, and the first medical center of the Chinese PLA General Hospital approved this current research. We retrospectively reviewed the data of those DLS patients who had undergone surgical treatments in the three hospitals ranging from June 2019 to August 2020.

### Inclusion and exclusion criteria

The inclusion criteria were as follows:

(i) Diagnosis of DLS; (ii), age ≥ 40 years; (iii), those underwent the surgical procedure of thoraco-lumbar fusion extending to the pelvis with instrumentations; and (iv), those with integrated data.

The exclusion criteria were as follows:

Patients (i) underwent spinal surgeries previously; (ii) suffered from other spinal disorders, such as tumor, tuberculosis or ankylosing spondylitis; (iii) had any disorders in lower extremities, involving hip or knee disorders; or (iv) had the differences ≥ 2 cm between the lower extremities.

### Surgical techniques

Those orthopedic surgeries were operated by three senior professors serving at the three different medical institutions. All of the participants collected in this current study were positioned prone after inducing general anesthesia. Then, somatosensory evoked potential and transcranial motor evoked potential were initiated. The surgical procedures of long sacroiliac fusion with instrumentations (titanium alloy screws and two-rod constructs) via posterior-only approach were performed. In addition, those surgical procedures of posterior lumbar inter-body fusion (PLIF) or transforaminal lumbar inter-body fusion (TLIF) were performed on such spinal stenosis segments.

### Radiographic evaluation

Long cassette standing radiographs were performed preoperatively, at the 3^rd^ month postoperative visit, and at the final follow-up in a weight-bearing position, in which those individuals placed the upper extremities on a support, and maintained the shoulders flexion at 30° forward and slight elbow flexion [19]. All of the radiographic measurements were performed by a dedicated team independent from the operating surgeons with the validated spine Software of Surgimap (version: 2.3.2.1; New York, NY) [20].

**Fig. 1:**
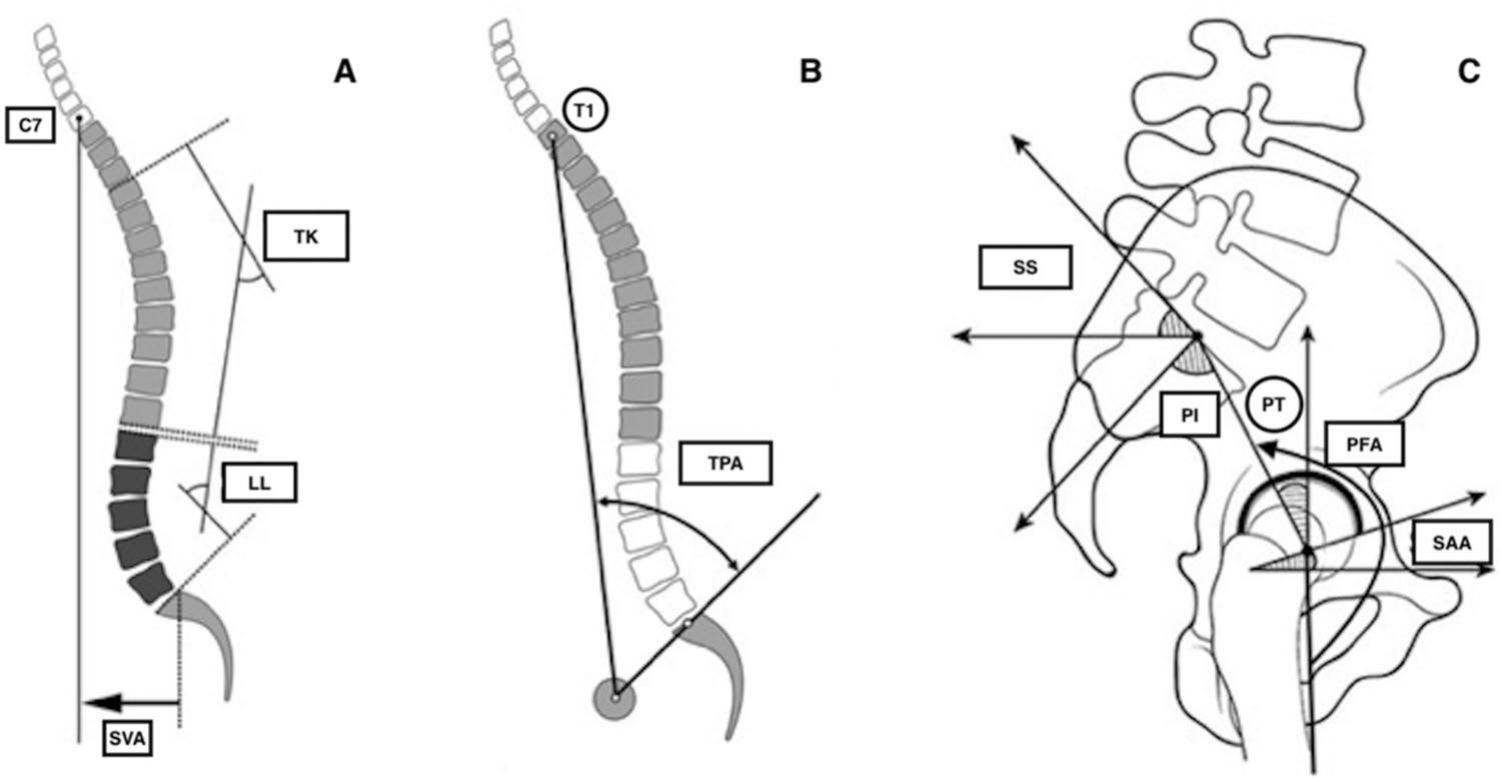
Schematic drawing of spinopelvic and hip parameters

Spinopelvic parameters concerned in this study include the thoracic kyphosis (TK), lumbar lordosis (LL), sagittal vertical axis (SVA), T1 pelvic angle (TPA), sacral slope (SS), pelvic tilt (PT), pelvic incidence (PI), sagittal acetabular anteversion (SAA), and pelvic femur angle (PFA), for which the measurement methods are listed in Table 1, and the schematic drawings are shown in Fig. 1A–C. The mismatch of pelvic incidence minus lumbar lordosis (PI–LL) was calculated subsequently.

**Table 1:** Radiographic parameters concerned in this current study

TK	The Cobb angle between the upper endplate of T4 and the lower endplate of T12
LL	The Cobb angle between the upper endplate of L1 and S1
SVA	the horizontal distance between the C7 plumb line and the posterosuperior corner of S1
TPA	The angle between the line from the axis of the femoral head to the centroid of T1 and the line from the axis of femoral head to the midpoint of the S1 endplate
SS	The angle between the sacral endplate and the horizontal line
PT	the angle between the line from the middle of the sacral plate to the middle of the hip axis and the vertical line
PI	The angle between the line perpendicular to the midpoint of the sacral plate and the line connecting this to the midpoint of the hip axis
SAA	The angle between the tangent line across the front and rear edge of the acetabulum and the horizontal line
PFA	The angle between the line from the middle of the sacral plate to the middle of the hip axis and the parallel line of the longitudinal axis of the femur

Kyphosis was recorded as positive (+), and lordosis as negative (–). Mismatch (PI–LL) were subsequently calculated by subtracting LL from PI. TK indicates thoracic kyphosis, *LL* Lumbar lordosis, *SVA* Sagittal vertical axis, *TPA* T1 pelvic angle, *SS* Sacral slope, *PT* Pelvic tilt, *PI* Pelvic incidence, *SAA* Sagittal acetabular anteversion, *PFA* Pelvic femur angle

### Quality-of-life (QoL) evaluation

The questionnaires of QoL in this current study included the short form 36 (SF-36) and the Oswestry disability index (ODI), which were recorded and documented at the pre-operation, the 3rd month, and the final follow-up postoperatively.

### Statistical analyses

Variables in this current study were recorded and expressed as mean ± standard deviation (SD). Comparisons of radiographic variables and QoL data among the pre-, post-operation, and the final follow-up were performed using the ANOVA test. Those changes of spinopelvic variables perioperatively were calculated (mean, standard deviation, and range). The Pearson correlation coefficient was calculated via linear regression analysis. The slope of the line of the best fit was used to predict the effect of LL correction on other spinopelvic parameters. All of those statistical analyses were performed with SPSS software (Mac version 26.0, IBM Corp.). Statistical difference was determined as *P* < 0.05.

## Results

There were 88 DLS patients (male/female: 21/67) concerned in this current study, including 22 cases from the affiliated hospital of Shandong University of Traditional Chinese Medicine, 10 cases from the affiliated hospital of Jining Medical University, and 56 cases from the Chinese PLA General Hospital. The mean age of all those subjects was 64.44 ± 8.37 years (ranging from 40 to 86 years) at the surgery. The average of follow-up duration was 28.24 ± 8.28 months (ranging from 24 to 40 months).

**Fig. 2:**
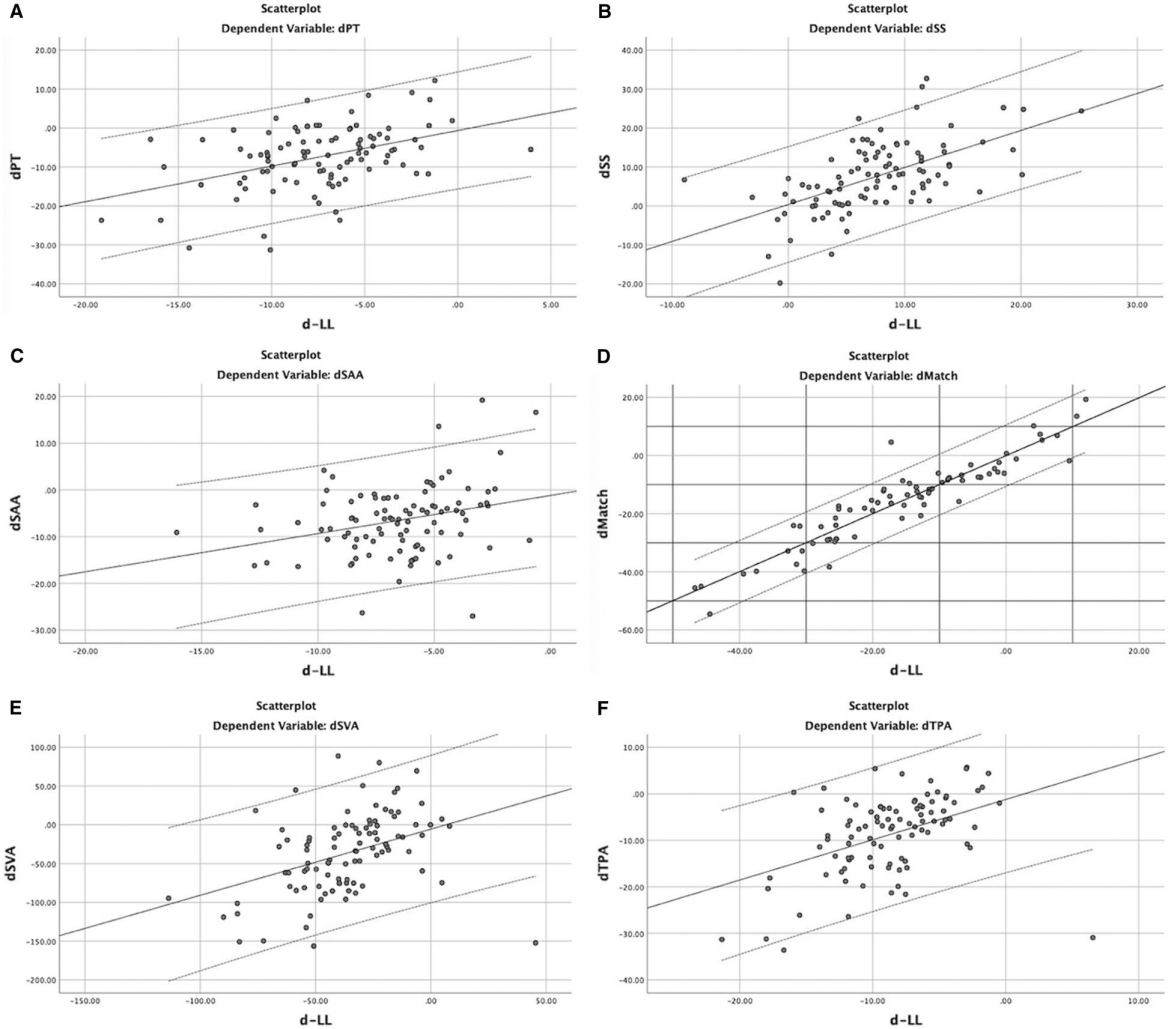
Scatterplots reveal the significant relationships between the correction in lumbar lordosis and the changes in other radiographic parameters. d-indicates the perioperative changes; *LL* lumbar lordosis, *TK* thoracic kyphosis, *SS* sacral slope, *PT* pelvic tilt, *PI–LL* the mismatch of pelvic incidence minus lumbar lordosis, *PFA* pelvic femur angle, *SAA* sagittal acetabular anteversion, *SVA* sagittal vertical axis, *TPA* T1 pelvic angle

There were significant improvements in the spinopelvic alignments (*P* < 0.001) and the QoL (*P* < 0.001) after surgical treatments (Table 2). Those perioperative changes in all radiographic parameters are listed in Table 3. The LL correction perioperatively correlated significantly (*P* < 0.001) with the changes in PT (*r* = −0.635), SS (*r* = 0.698), TPA (*r* = −0.782), SVA (*r* = −0.591) and PI–LL (*r* = −0.936), respectively. Moreover, linear-regression analyses revealed that 1° of increase in LL occurred with −4.01° in PT (*r*^*2*^ = 0.404), −4.86° in TPA (*r*^*2*^ = 0.612), 2.08° in PI–LL (*r*^*2*^ = 0.876), 2.62° in SS (*r*^*2*^ = 0.488), and −15.74 mm in SVA (*r*^*2*^ = 0.349). The details are listed in Table 4, and shown in Fig. 2. Although the LL correction correlated weakly with the changes of TK (*r* = 0.259, *P* = 0.01) and SAA (*r* = −0.359, *P* < 0.001), and even independently with the reduction in PFA (*r* = 0.12; *P* = 0.299), the TK, SAA and PFA in all subjects improved significantly after surgery (Tables 2 and 4).

**Table 2:** Comparisons of all of the radiographic parameters and HRQOL data before and after surgery

Variables	Pre-operation	Post-operation	Final follow-up	*P*
TK	16.33 ± 12.42	21.61 ± 9.77	28.78 ± 11.46	< 0.001
LL	23.18 ± 18.36	38.60 ± 12.47	40.21 ± 11.70	< 0.001
SS	23.47 ± 12.03	30.04 ± 10.48	28.86 ± 11.82	< 0.001
PT	23.31 ± 11.07	16.83 ± 9.20	19.32 ± 10.21	< 0.001
PI	46.91 ± 11.47	46.98 ± 11.72	47.07 ± 11.20	0.901
SAA	44.42 ± 8.20	38.45 ± 7.57	35.56 ± 8.27	< 0.001
PFA	198.58 ± 10.67	190.99 ± 9.79	194.12 ± 12.01	< 0.001
PI–LL	23.72 ± 18.12	8.38 ± 12.57	9.21 ± 13.72	< 0.001
SVA	44.73 ± 49.94	11.16 ± 33.13	22.63 ± 40.17	< 0.001
TPA	21.69 ± 11.92	14.39 ± 9.03	16.21 ± 10.39	< 0.001
ODI	47.80 ± 15.67	21.10 ± 13.20	30.76 ± 14.88	< 0.001
PCS	29.9 ± 8.27	42.80 ± 9.81	36.23 ± 8.88	< 0.001
MCS	50.10 ± 9.93	54.88 ± 10.02	48.21 ± 8.95	< 0.001

The values were given as the mean and the standard deviation.

TK indicates thoracic kyphosis, *SS* Sacral slope, *PT* Pelvic tilt, *TPA* T1 pelvic angle, *SVA* Sagittal vertical axis, *PI–LL* Mismatch pelvic incidence minus lumbar lordosis, *SAA* Sagittal acetabular anteversion, *PFA* Pelvic femur angle, *HRQOL* Health-related questionnaires of life, *ODI* Oswestry disability index, *PCS* Physical component score of SF-36, *MCS* Mental component score of SF-36

**Table 3:** Perioperative changes in spinopelvic parameters

Spinopelvic parameters	Perioperative changes^a^
Lumbar lordosis (°)	15.42 ± 13.52 (−13.30 to + 49.40)
Thoracic kyphosis (°)	5.27 ± 9.09 (−18.20 to + 27.00)
T1 pelvic angle (°)	−7.30 ± 7.32 (−33.60 to + 5.70)
Sagittal vertical axis (mm)	−33.57 ± 47.96 (−156.30 to + 80.10)
Pelvic incidence minus lumbar lordosis (°)	−15.34 ± 12.52 (−54.50 to + 13.50)
Sacral slope (°)	6.57 ± 8.47 (−19.80 to + 32.70)
Pelvic tilt (°)	−6.48 ± 7.40 (−30.80 to + 12.20)
Sagittal acetabular anteversion (°)	−6.25 ± 7.32 (−27.00 to + 19.20)
Pelvic femur angle (°)	−7.59 ± 6.66 (−25.5 to + 7.30)

^a^The values were given as the mean and the standard deviation, with the range in parentheses

**Table 4:** Correlations between changes perioperatively in ll and spinopelvic parameters

Correction in lumbar lordosis	Changes in spinopelvic parameters							
	TK	SS	PT	TPA	SVA	PI–LL	SAA	PFA
Bivariable correlation^a^								
r	0.259	0.698	−0.635	−0.782	−0.591	0.936	−0.359	0.120
P value	0.01	< 0.001	< 0.001	< 0.001	< 0.001	< 0.001	< 0.001	0.299
Linear regression^a^								
r^2^	N/A	0.488	0.404	0.612	0.349	0.876	N/A	N/A
P value	N/A	< 0.001	< 0.001	< 0.001	< 0.001	< 0.001	N/A	N/A
Coefficient^b^	N/A	0.42	−0.342	−0.432	−1.755	0.911	N/A	N/A
Standard error	N/A	0.065	0.063	0.052	0.361	0.056	N/A	N/A
P value	N/A	< 0.001	< 0.001	< 0.001	< 0.001	< 0.001	N/A	N/A

TK indicates thoracic kyphosis; *SS* Sacral slope, *PT* Pelvic tilt, *TPA*, T1 pelvic angle, *SVA* Sagittal vertical axis, *PI–LL* Mismatch of pelvic incidence minus lumbar lordosis, *SAA* Sagittal acetabular anteversion, *PFA* Pelvic femur angle

^a^Linear regression analysis showed significant correlation changes in all spinopelvic parameters and lumbar lordosis; N/A = not applicable; ^b^ The coefficient refers to the 1° increase in lumbar lordosis that would result in changes in each parameter.

Of 88 subjects, 76 individuals (86.4%) had severe sagittal decompensation at the pre-operation, suffering from PT > 25°, SVA > 50 mm or PI–LL > 20° [21, 22]. Postoperatively, there were still 11 cases (12.5%) with PI–LL > 20° and 8 cases (9.1%) with PI–LL < 10°. Patients showing proximal junctional kyphosis (PJK) [23] equal to 21 (23.9%) at the final follow-up. Of those, ten cases (11.4%) with PI–LL > 20° or PI–LL < 10° at the 3^rd^ month postoperatively developed symptomatic PJK (two cases) or proximal junctional failure (PJF) (eight cases) during follow-up.

**Fig. 3:**
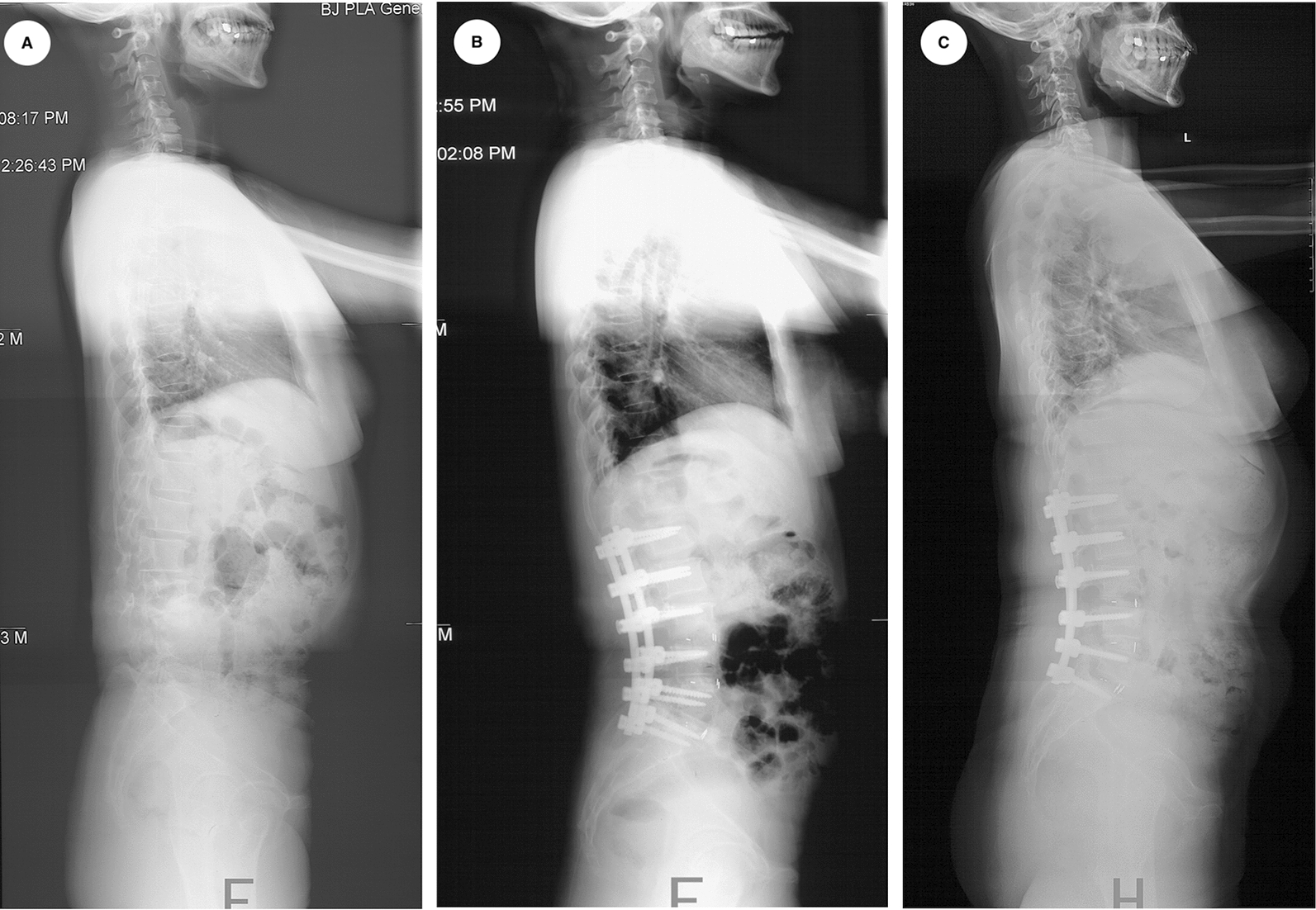
A 68-year-old male DLS patient underwent lumbar fusion surgery (L1–S1). Radiographs show the changes in spinopelvic parameters, preoperative TK, LL, PT, SS, PI, PFA, SAA, SVA and TPA were 6.6°, −15.8°, 26.6°, 25.4°, 52.0°, 200.0°, 36.0°, 2.6 mm and 17.7°, respectively (**A**). Postoperatively, those variables were 14.7° for TK, −41.1° for LL, 18.8° for PT, 34.1° for SS, 52.9° for PI, 185.4° for PFA, 35.9° for SAA, −27.3 mm for SVA, and 8.7° for TPA (**B**). At the final follow-up, those variables were 15.8° for TK, −41.2° for LL, 18.9° for PT, 38.9° for SS, 57.8° for PI, 189.6° for PFA, 36.2° for SAA, 17.8 mm for SVA, and 14.6° for TPA (**C**). The PI–LL was 36.2°, 11.8° and 16.6° at the pre-, post-operation and the final follow-up, respectively

**Fig. 4:**
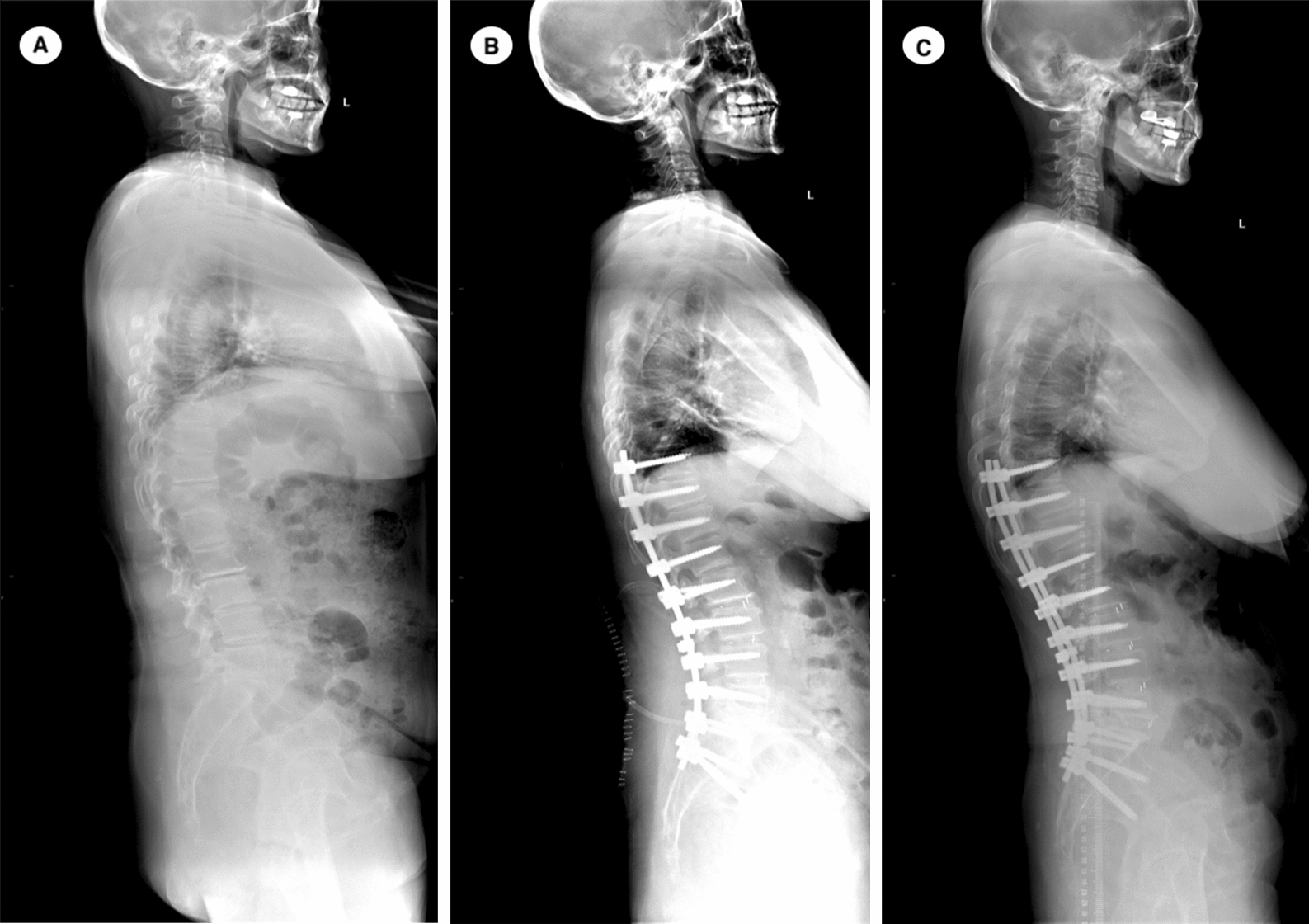
A 58-year-old female DLS patient underwent thoracolumbar fusion surgery (T10–S2). Radiographs show the changes in spinopelvic parameters, preoperative TK, LL, PT, SS, PI, PFA, SAA, SVA and TPA were 16.9°, −16.9°, 27.5°, 4.2°, 31.7°, 197.2°, 51.0°, −4.3 mm and 19°, respectively (**A**). Postoperatively, those variables were 37.6° for TK, -35.8° for LL, 20° for PT, 15.3° for SS, 35.3° for PI, 193.7° for PFA, 49.2° for SAA, −21.5 mm for SVA, and 14° for TPA (**B**). The PI–LL postoperatively was -0.5° at the post-operation. The patient has a significant upright posture after the surgery (**B**); however, PJF developed at the 18^th^ month during follow-up **(C)**

**Fig. 5:**
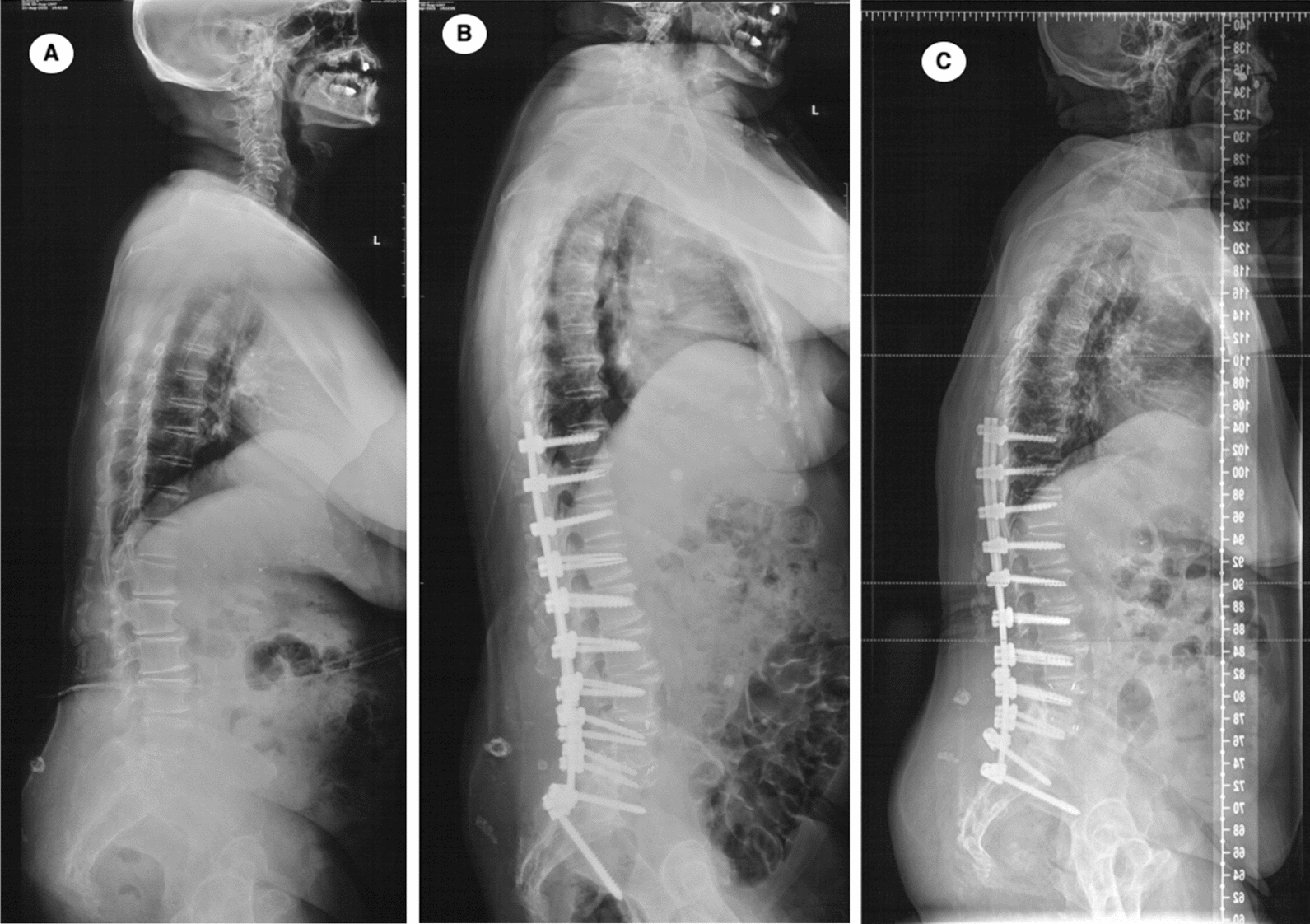
A 68-year-old female DLS patient underwent thoracolumbar fusion (T10–S2) surgery. Radiographs show the changes in spinopelvic parameters, preoperative TK, LL, PT, SS, PI, PFA, SAA, SVA and TPA were 11.3°, −7.3°, 27.3°, 20.9°, 48.2°, 206.4°, 52.0°, 90.7 mm and 29°, respectively (**A**). Postoperatively, those variables were 20.3° for TK, −25.1° for LL, 24.7° for PT, 21.8° for SS, 49.5° for PI, 193.8° for PFA, 50.3° for SAA, 15.3 mm for SVA, and 26.2° for TPA (**B**). The PI–LL was 24.8° at the post-operation. However, the patient had intermittent back pain after surgery, and PJK developed at the 4^th^ month during follow-up **(C)**

Three representative DLS patients underwent orthopedic surgery are shown in Fig. 3, 4 and 5.

## Discussion

It is well-known that loss of lumbar lordosis (LL) probably be the initiating pathology in degenerative lumbar scoliosis (DLS) [2]. The full-body alignment affecting quality of life in DLS patients would be deteriorating subsequently. In our current study, 76 patients (86.4%) suffered from significant full-spinal imbalance at the pre-operation, having PT > 25°, SVA > 50 mm, or PI–LL > 20° [21, 22]. The spinopelvic alignments in all subjects improved significantly after thoracolumbar fusion surgery. Moreover, linear-regression analyses showed that the LL correction correlated significantly to the changes in SVA and TPA, respectively. An increase of 1° in LL may correlate to a reduction of 4.86° for TPA (*r*^*2*^ = 0.612) and a reduction of 15.74 mm for SVA (*r*^*2*^ = 0.349), respectively. As a result, the restoration of LL is essential for spinopelvic realignments in DLS patients. Radiographic parameters involving PI–LL, SVA, and TPA have been demonstrated to be significantly associated with QoL in patients with adult spinal deformity [24–28]. Moreover, a surgical target of 10–20° and less than 50 mm for TPA [28, 29] and SVA [22] was suggested, respectively. Then, the results in our current study could provide the orthopedic algorithms for spinal surgeons in management of DLS.

PI–LL, representing the match between pelvis and lumbar spine, was proposed to be a surgical target of 10–20° for adult scoliosis in recent studies [30, 31]. In our current study, there were still 11 cases (12.5%) with PI–LL > 20° and 8 cases (9.1%) with PI–LL < 10° after surgery. Although there were only four patients with the abnormal PI–LL according to the criteria proposed by Lafage et al. [32], all patients developing PJK during follow-up had the PI–LL > 20° or < 10°. We speculate that such patients may have overcorrection (PI–LL < 10°) or under-correction (PI–LL > 20°) in LL, respectively. The proximal junctional stress may increase significantly, which will result in proximal junctional diseases happening subsequently during follow-up. The patient shown in Fig. 4 had significant restoration in the spinopelvic alignments after a overcorrection in the LL, with the PI–LL = −0.5° postoperatively; however, the patient suffered from PJF at the 18th month after surgery. Conversely, the case shown in Fig. 5 with the PI–LL = 24.8° postoperatively probably has under-correction in the LL, and the PJK developed at the 4th month after surgery. Linear regression analysis showed that an increase of 1° in LL may correlate with an increase of 2.08° in PI–LL (*r*^*2*^ = 0.876), which may help spinal surgeons to reduce the incidence of PJK/PJF in management of DLS.

The pelvis probably plays an essential role in keeping sagittal balance both in standing and sitting positions, which were demonstrated in previous studies [33, 34]. PT was recognized as a reservoir to compensate the full-spinal balance, which correlated significantly with QoL in DLS patients [8, 21, 22, 24], and should be no more than 20° [22, 35]. In this current study, we observed pelvic rotation backward significantly in almost all patients at the pre-operation, and those pelvic parameters improved significantly after orthopedic surgery. The LL correction correlated significantly with the changes in PT (*r* = −0.635) and SS (*r* = 0.698), respectively. In addition, linear regression analyses illustrated that 1° of LL correction occurred with the changes of 4.01° in PT (*r*^*2*^ = 0.404) and 2.62° in SS (*r*^*2*^ = 0.488), which may help spinal surgeons to predict the PT postoperatively in DLS.

Hip joints extension is another important compensatory mechanism in DLS patients with full-spinal imbalance on sagittal plane. However, the abnormal acetabular anteversion postoperatively may increase the incidence of mechanism complications in adult patients underwent long-fusion surgery [12]. Therefore, it is important for hip and spine surgeons to clarify the relationships between LL correction and changes in hip variables in evaluation and management of patients suffering from hip–spine syndrome. Masquefa et al. [36] illustrated the significant relationships between LL correction and changes in acetabular anteversion (*r* = 0.34) in DLS patients underwent the surgical procedure of long-fusion with pedicle subtraction osteotomies. In our current study, hip variables including SAA and PFA had significant improvements after surgery; however, the LL correction correlated mildly to the changes in SAA (*r* = −0.359). As a result, those relationships between the LL correction and the changes in hip variables and PT in our current study would bridge the gap between hip and spine surgeons in the management of DLS patients coexisting with hip disorders.

It was reported that structural changing of LL may affect the shape of thoracic kyphosis and the orientation of the pelvis [37]. In our current study, all of those participants had significant changes in thoracic kyphosis (TK) and pelvic femur angle (PFA) after thoracolumbar fusion surgery. Interestingly, the changes in TK (*r* = 0.259) and PFA (*r* = 0.12) were independent of the LL correction. Furthermore, the mean value of TK, TPA and SVA was increasing during the follow-up. The serious degeneration in paraspinal muscles may be the causative factor in such DLS patients, which has been proven to be associated with a various of lumbar disorders and diseases [38, 39]. Moreover, the erector spinae degenerated diffusely and correlated with sagittal imbalance [39]. As a result, we propose that the TK probably to be remodeled themselves due to the seriously degenerative paraspinal muscles, which can keep the upright posture effectively in those DLS patients underwent long-fusion surgery. However, it is regrettable that those variables of paraspinal muscle were not collected initially in our current study.

Limitations in our current study should be mentioned. First, we, respectively, reviewed the DLS patients treated in three medical centers; however, the sample size was still limited because of the strict inclusion criteria. Second, although pelvic fusion in all patients can decrease the errors in results of hip parameters resulted by the dynamic lumbosacral joints, those measurements of hip variables were performed on radiographs of patients with spinal deformity rather than standard pelvic radiographs. Third, according to those results reported in previous studies, the thoracic spine and hip variables changes significantly after surgery may be due to the degeneration in paraspinal muscles; however, we did not collect those variables of paraspinal muscles initially. Finally, the retrospective design may undermine the confidence level of this current study. However, the results in our current study illustrated the significant relationships between the perioperative correction in lumbar lordosis and the changes in other spinopelvic parameters, even with those limitations mentioned above.

## Conclusions

The spine–pelvic–hip alignments will improve significantly in patients with degenerative lumbar scoliosis who had undergone the surgical procedure of long sacroiliac fusion with instrumentations. The significant relationships between the LL correction and the changes in spinopelvic parameters would provide the surgical algorithms for spinal surgeons in management of DLS. The thoracic spine and lower extremities being independently with the LL correction probably be remodeled themselves to keep the full-body balance after correction surgery.

## Data Availability

All data generated during this study are available from the corresponding author on reasonable request. There was no data published previously.
